# Diagnostic Value of Neutrophil-to-Lymphocyte Ratio and Platelet-to-Lymphocyte Ratio in Crohn's Disease

**DOI:** 10.1155/2017/3526460

**Published:** 2017-07-17

**Authors:** Jue-Rong Feng, Xiao Qiu, Fan Wang, Peng-Fei Chen, Qian Gao, Ya-Nan Peng, Xue Lin, Qing Liu, Jing Liu, Qiu Zhao, Jin Li

**Affiliations:** ^1^Department of Gastroenterology, Zhongnan Hospital of Wuhan University, Wuhan, China; ^2^Hubei Clinical Center & Key Laboratory of Intestinal & Colorectal Diseases, Wuhan, China

## Abstract

The aim of this study is to investigate the diagnostic efficacy of neutrophil-to-lymphocyte ratio (NLR), neutrophil-to-monocyte ratio (NMR), lymphocyte-to-monocyte ratio (LMR), and platelet-to-lymphocyte ratio (PLR) in patients with Crohn's disease (CD) and non-CD controls. These ratios were all derived from complete blood counts. Two hundred and six participants including CD inpatients and non-CD controls were retrospectively enrolled. We found statistically higher NLR and PLR and lower LMR in CD patients than in non-CD controls (all *P* < 0.01). However, NMR was not different between the two groups (*P* = 0.18). In addition, NLR, PLR, and LMR were associated with CRP and ESR. Optimal cutoffs for NLR and PLR were 2.72 (sensitivity: 68.3%, specificity: 75.9%, and overall accuracy: 70.1%) and 132.88 (sensitivity: 76.7%, specificity: 84.8%, and overall accuracy: 80.8%), respectively. In conclusion, the NLR and PLR might be effective, readily available, and low-cost biomarkers for differentiating CD patients from non-CD controls.

## 1. Introduction

Crohn's disease (CD) is characterized by chronic, relapsing/remitting inflammation in any section of the gastrointestinal tract [1]. The aetiology of CD is not yet fully understood, but many studies have shown an increase in the prevalence of CD in many countries around the world [2, 3].

Biomarkers in CD can aid in the diagnosis and monitoring of disease activity in clinical practice. Although endoscopy continues to be the gold standard for diagnosis of CD and monitoring disease activity, it is an invasive and inconvenient examination and it may not be appropriate in the setting of severe disease. However, to date, no ideal biomarker has been identified to assess and manage CD. For example, despite the fact that erythrocyte sedimentation rate (ESR) and C-reactive protein (CRP) can be altered in active CD, they are nonspecific markers that can be altered in various inflammatory processes [4–6]. Fecal calprotectin (FC) and lactoferrin can indicate disease activity and predict relapse of CD, but a further endoscopy was needed for evaluation [7–10]. In addition, fecal S100A12, a calcium-binding calgranulin protein, can distinguish CD from irritable bowel syndrome [11]. Despite the data demonstrating the value of these biomarkers, none of them can replace the necessity to undergo endoscopy to assess the intestinal condition.

Complete blood counts including leukocyte subtypes are commonly examined during admission of CD patients. The neutrophil-to-lymphocyte ratio (NLR), neutrophil-to-monocyte ratio (NMR), lymphocyte-to-monocyte ratio (LMR), and platelet-to-lymphocyte (PLR) are also easily calculated from the absolute neutrophil count, the absolute lymphocyte count, the absolute monocyte count, and the absolute platelet count, which can be obtained from just a single complete blood count test. The NLR as a novel biomarker was used to diagnose ulcerative colitis (UC) and predict a patient's overall disease course [12–15]. The other ratios have not yet been evaluated in the context of CD [16]. Therefore, the aim of this study was to assess the utility of NLR, NMR, PLR, and LMR in patients with CD in comparison to healthy controls without CD.

## 2. Material and Methods

### 2.1. Participants

This retrospective study reviewed the data from inpatients with active CD and healthy controls from electronic medical records (EMR) in the Department of Gastroenterology, Zhongnan Hospital of Wuhan University, between May 2014 and June 2016. Non-CD controls, who received annual health checkups during the study period, were age- and gender-matched with one CD patient randomly selected from EMR. The diagnosis of CD patients was based on standard clinical, radiological, endoscopic, and histological records [17]. The criteria for inclusion in the study group were as follows: (1) subjects underwent blood sampling collection for complete blood counts at the time of admission and prior to the commencement of any therapy and (2) patients were newly diagnosed with CD. Patients were excluded if they had an acute infectious disease or other underlying diseases (such as colorectal cancer, hepatocellular cancer, and multiple myeloma) that could influence the ratios of interest.

Patients' age, gender, and other medical history were all reviewed and collected from EMR. The detailed results of complete blood count testing were obtained for both groups. The NLR and PLR were calculated by dividing the absolute neutrophil count by the absolute lymphocyte count and dividing the absolute platelet count by the absolute lymphocyte count, respectively. In addition, LMR and NMR were calculated by dividing the absolute lymphocyte count by the absolute monocyte count and the absolute neutrophil count by the absolute monocyte count, respectively.

### 2.2. Statistical Analysis

Comparisons between groups were performed using the Student *t*-test or Mann–Whitney nonparametric tests according to whether it met normal distribution. Data were verified by the Student *t*-test for normal distribution. Then, Spearman's correlation analyses were conducted between the ratios and inflammatory biomarkers such as CRP and ESR. Receiver operating characteristic (ROC) curve analyses were conducted to assess the performance of each biomarker in differentiating CD from non-CD controls. The ROCs were produced by plotting the sensitivity value against the false-positive rate (1 − specificity). Accuracy of each biomarker was measured by the area under the ROC curve. The predictive value of each ratio was assessed by calculating the area under the curve (AUC) and estimated the optimal cutoff value based on the maximum Youden index. The overall accuracy was also calculated by dividing the sum of true-positive and true-negative tests by all tests. These analyses were conducted using SPSS 17.0 software (SPSS Inc., Chicago, IL) by considering a two-tailed *P* < 0.05 significantly different.

## 3. Results

### 3.1. Subject Characteristics

A total of 527 participants including 207 CD inpatients and 320 non-CD controls were reviewed in EMR during the study period. After consideration of the inclusion and exclusion criteria, 103 participants in each group were identified (Table 1). There was no significant difference in gender and age between the CD group and the control group (all *P* > 0.05).

### 3.2. Comparisons and Correlations of Ratios between the Two Groups

There were not any differences between NMR, WBC, and neutrophil or monocyte between the both groups (*P* = 0.18; 0.17, 0.79, and 0.22, resp.). The NLRs of CD patients and non-CD controls were 2.95 and 2.33, respectively (*P* < 0.01). The other ratios (PLR and LMR) were markedly elevated in CD patients compared to non-CD controls (Table 1).

Spearman's correlation analyses indicated positive correlations between NLR and CRP (*r* = 0.43, *P* < 0.01) and ESR (*r* = 0.39, *P* < 0.01) in CD patients. In addition, PLR was positively correlated with CRP (*r* = 0.29, *P* < 0.01) and ESR (*r* = 0.57, *P* < 0.01) in CD patients. In contrast, LMR was negatively related to CRP (*r* = −0.39, *P* < 0.01) and ESR (*r* = −0.33, *P* < 0.01) in CD patients (Table 2).

### 3.3. Optimal Cutoffs of NLR and PLR

**Figure 1:**
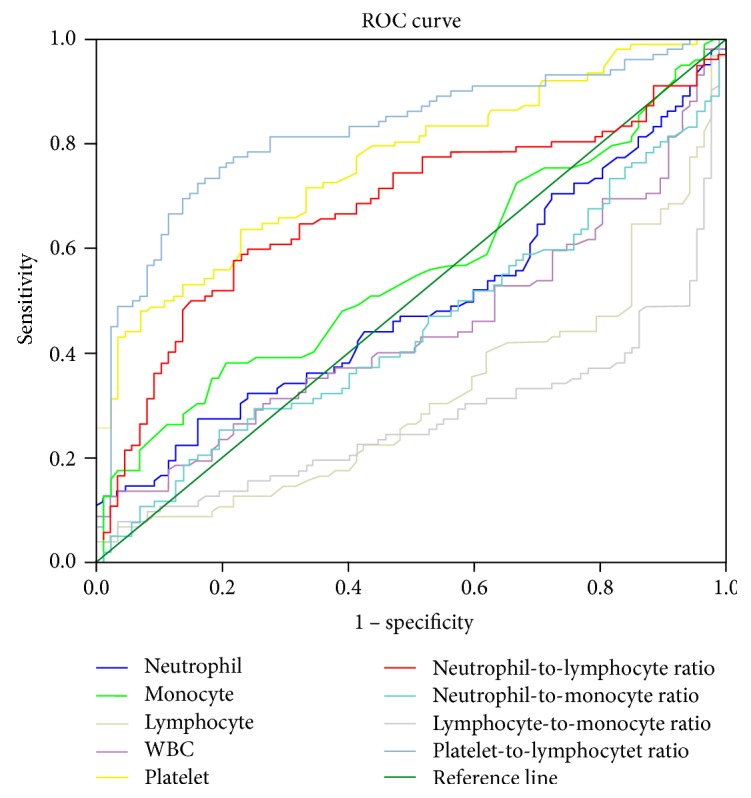
Receiver operating characteristic (ROC) curve of multiple blood biomarkers in 103 patients with Crohn's disease and 103 healthy control subjects.

The ROC analyses were performed to establish cutoff points for NLR, LMR, and PLR. The results suggested that NLR (AUC: 0.74, 95%CI: 0.65–0.83) and PLR (AUC: 0.86, 95%CI: 0.80–0.92) were useful in differentiating the patients with CD from controls (Figure 1 and Table 3). For differentiating CD patients from non-CD controls, optimal cutoffs of NLR and PLR based on the largest Youden index were 2.72 (sensitivity: 68.3%, specificity: 75.9%, and overall accuracy: 70.1%) and 132.88 (sensitivity: 76.7%, specificity: 84.8%, and overall accuracy: 80.8%), respectively. However, the AUC of LMR was 0.25, indicating low test utility (Table 3).

## 4. Discussion

In this retrospective study, data mining was performed for more clinical value in the complete blood count test, which is one of the most frequently requested tests in patients with CD. Our findings revealed that elevated NLR and PLR and decreased LMR, which were derived from complete blood counts, were significantly different between CD patients and non-CD controls. Furthermore, these ratios correlated strongly with CRP and ESR. ROC analyses revealed that a cutoff of 2.72 for NLR and a cutoff of 132.88 for PLR had high sensitivity, specificity, and predictive values to differentiate CD patients from non-CD controls. In addition, total WBC, neutrophil counts, and monocyte counts were not significantly different between the two groups, which indicated that NLR and PLR were more sensitive and meaningful than neutrophil and monocyte counts alone.

To our knowledge, WBC, CRP, and ESR are the most commonly used inflammatory indicators in routine clinical practice for CD patients. These parameters can change with the degree of the inflammatory status of CD. Both NLR and PLR are positively correlated with ESR and CRP in the current study. These two ratios are simple and inexpensive examinations of a systemic inflammatory biomarker that correlates with prognosis in distinct diseases. The NLR has been generally investigated in inflammatory and neoplastic diseases, such as ST-segment elevation myocardial infarction (STEMI), ulcerative colitis, colorectal cancer, hepatocellular cancer, multiple myeloma, and type 2 diabetes, as a prognostic index [14, 18–23]. Therefore, CD patient with an already complicated disease was excluded in advance to avoid this as a confounding factor. In addition to NLR, PLR has been reported as a novel inflammatory biomarker of promise for screening and predicting prognosis for acute pulmonary embolism, psoriasis, ovarian cancer, and colorectal cancer [24–27].

To date, only two studies have assessed the value of NLR in CD. One study showed that CD patients with high NLR before infliximab therapy had a lower NLR after 52 weeks of therapy compared with controls. The authors suggested that NLR may be a useful predictor of response to infliximab and could be utilized to optimize the infliximab dosing schedule [28]. Another study verified that elevated NLR could differentiate CD patients from non-CD controls [16], which was in line with our findings. The elevated NLR and PLR stem from both a reduction in the lymphocyte count and an increase in the neutrophil count and the platelet count. Our results showed similar findings that the quantity of lymphocyte was reduced in CD [29]. Elevated leukocytes including neutrophils reflect systemic inflammation and contribute to innate and adaptive immune responses [30]. During infectious and noninfectious inflammatory disorders, neutrophils are generated in the bone marrow and migrate to the inflamed tissues following the release of proinflammatory cytokines and chemokines. The study by Catarzi et al. [31] found that the apoptosis of polymorphonuclear neutrophils (PMN) was delayed and that the life of circulating PMN was prolonged, which can be responsible for their excessive migration to inflamed intestinal sites [31]. Excessive activation of leukocytes and aberrant innate or adaptive immunity are known to be involved in the pathogenesis of CD [32].

The limitations of this study should be pointed out. The study utilized a retrospective case-control design with patients diagnosed with CD matched to healthy control subjects. ESR and CRP data were not available in the control subjects, as these tests were not part in routine health checks.

## 5. Conclusion

A comprehensive evaluation of neutrophil, lymphocyte, and monocyte and their ratios from complete blood counts was assessed in CD patients. NLR and PLR appear to be promising inflammatory biomarkers in CD. These biomarkers have the advantage of being routinely available, noninvasive, and low-cost. Future prospective studies are now required to further evaluate the utility of these biomarkers in patients with CD.

## Figures and Tables

**Table 1:** General characteristics of 103 patients with Crohn's disease (CD) and 103 healthy control subjects.

	CD group	Control group	*P* value	Normal range
Age (year)	31.84 ± 11.98	35.06 ± 12.28	0.06	—
Gender (F/M)	31/72	22/65	0.46	—
WBC	5.79 (2.79–22.43)	6.34 (2.34–9.86)	0.17	4~10 × 10^9^
Neutrophil	3.49 (0.92–21.55)	3.69 (1.31–6.50)	0.79	1.2~6.8 × 10^9^
Lymphocyte	1.44 ± 0.74	1.83 ± 0.56	***<*** *0.01*	0.8~4.0 × 10^9^
Monocyte	0.48 (0.19–3.14)	0.45 (0.14–7.2)	0.22	0.3~0.8 × 10^9^
Platelet	237.00 (112–602)	168.00 (36–317)	***<*** *0.01*	100–300 × 10^9^
Neutrophil-to-lymphocyte ratio	2.95 (0.23–46.85)	2.33 (0.95–10.17)	***<*** *0.01*	—
Neutrophil-to-monocyte ratio	7.56 (1–546)	8.25 (0.62–18.73)	0.18	—
Lymphocyte-to-monocyte ratio	3.02 ± 1.91	4.1 ± 1.31	***<*** *0.01*	—
Platelet-to-lymphocyte ratio	171.61 (54.44–850)	93.49 (34.23–500)	***<*** *0.01*	—

Values are median (minimum, maximum) if not normally distributed. Values are mean ± SD if normally distributed. The italics indicates comparisons with significant differences.

**Table 2:** Spearman's correlation coefficients of neutrophil-to-lymphocyte ratio (NLR) and platelet-to-lymphocyte ratio (PLR) with other inflammatory markers in Crohn's disease (CD) patients.

	NLR		PLR		LMR	
	*r*	*P*	*r*	*P*	*r*	*P*
CRP	0.43	*<0.01*	0.29	*<0.01*	−0.39	*<0.01*
ESR	0.39	*<0.01*	0.57	*<0.01*	−0.33	*<0.01*

The italics indicates correlations with significant differences.

**Table 3:** Accuracy of neutrophil-to-lymphocyte ratio (NLR) and other inflammatory markers in differentiating Crohn's disease (CD) patients from healthy controls.

Parameters	CD group versus control group						
	AUCs	Cutoffs	Sensitivity (%)	Specificity (%)	PPV (%)	NPV (%)	Overall accuracy (%)
Lymphocyte	0.28	2.69	5.00%	97.50%	66.67%	50.65%	51.25%
Platelet	0.77	242.50	53.30%	91.10%	85.69%	66.11%	72.20%
Neutrophil-to-lymphocyte ratio	*0.74*	*2.72*	*68.30%*	*75.90%*	*73.92%*	*70.54%*	*72.10%*
Neutrophil-to-monocyte ratio	0.47	10.61	31.70%	82.30%	64.17%	54.65%	57.00%
Lymphocyte-to-monocyte ratio	0.25	6.57	6.70%	97.50%	72.83%	51.10%	52.10%
Platelet-to-lymphocyte ratio	*0.86*	*132.88*	*76.70%*	*84.80%*	*83.46%*	*78.45%*	*80.75%*

The italics indicates comparisons with significant differences. PPV: positive predictive value; NPV: negative predictive value.
